# Synthesis of Terpyridine End-Modified Polystyrenes through ATRP for Facile Construction of Metallo-Supramolecular P3HT-*b*-PS Diblock Copolymers

**DOI:** 10.3390/polym12122842

**Published:** 2020-11-29

**Authors:** Tsung-Han Tu, Yi-Tsu Chan

**Affiliations:** Department of Chemistry, National Taiwan University, Taipei 10617, Taiwan; d02223101@ntu.edu.tw

**Keywords:** metallo-initiator, terpyridine, heteroleptic complexation, self-assembly, metallo-supramolecular copolymer

## Abstract

Complementary complexation between 2,2′:6′,2″-terpyridine (tpy) and 6,6″-dianthracenyl-substituted tpy in the presence of Zn(II) ions provided an efficient strategy for construction of metallo-supramolecular diblock copolymers. To synthesize well-defined tpy-modified polystyrenes (PSs), an Fe(II) bis(tpy) complex bearing α-bromoester as a metallo-initiator was applied to atom transfer radical polymerization (ATRP) to avoid poisoning the Cu(I) catalyst. Subsequently, a series of tpy-functionalized PSs was obtained after the decomplexation of <tpy-Fe(II)-tpy> junction by tetrakis(triethylammonium) ethylenediaminetetraacetate (TEA-EDTA) under mild conditions. The metallo-supramolecular poly(3-hexylthiophene) (P3HT)-*block*-PS diblock copolymers were prepared by simply mixing the corresponding terminally tpy-modified homopolymers with Zn(II) ions, and further characterized by ^1^H NMR and diffusion ordered spectroscopy (DOSY) experiments. The approach using metallo-initiators for ATRP offers an opportunity to construct tpy-functionalized polymers with controllable molecular weights and low polydispersities. Through the spontaneous heteroleptic complexation, a variety of metallo-supramolecular diblock copolymers with tunable block ratios can be easily constructed.

## 1. Introduction

Metallo-supramolecular block copolymers are generated through metal–ligand coordination of ligand end-modified homopolymers with proper metal ions [1]. Various ligands have been utilized in construction of diverse copolymers, such as 2,2′-bipyridine (bpy) [2,3,4], 2,2′:6′,2″-terpyridine (tpy) [5,6,7,8,9,10,11,12,13], and pincer-type ligands [14,15,16,17,18]. Among them, bis(tpy) complexes are commonly used for preparation of diblock copolymers in a stepwise manner [6,7,8,9,10,11,12]. However, the challenge remains to prevent unwanted homoleptic complexation and tedious purification processes. Recently, we demonstrated that the spontaneous heteroleptic complexation between unsubstituted tpy and 6,6″-dianthracenyl-substituted tpy could be achieved upon the addition of Zn(II) ions under ambient conditions, and was further applied to the construction of poly(3-hexylthiophene)-*block*-poly(ethylene oxide) (P3HT-*b*-PEO) copolymers [19]. The complementary ligand pairing indeed provides an efficient approach for synthesis of metallo-supramolecular diblock copolymers.

In order to incorporate tpy motifs into a wide variety of well-defined polymers, the tpy-modified chain-transfer agents or initiators have been feasibly utilized in controlled polymerizations, such as reversible addition-fragmentation chain transfer (RAFT) [20,21,22], nitroxide-mediated radical polymerization (NMP) [23,24,25,26], and ring-opening polymerization (ROP) [27,28,29]. However, since the atom transfer radical polymerization (ATRP) is often mediated by copper catalysts, the presence of uncomplexed tpy ligands could adversely affect the copper catalyst during the ATRP reaction [30,31]. To attenuate the ligand interference, bpy ligands have been successfully introduced into a polymeric structure via the ATRP initiated by bpy-based metallo-initiators [32,33,34,35,36,37,38,39,40]. For example, the Ru(II), Fe(II), and Zn(II) tris(bpy) complexes bearing chloromethylpyridine and *α*-bromoester have been employed in the ATRP of styrene and methyl methacrylate [41,42], where the use of metallo-initiators with coordinated bpy ligands efficiently prevented poisoning of copper catalysts. Therefore, inspired by the aforementioned researches, herein the use of an Fe(II) bis(tpy) complex as metallo-initiators in ATRP is realized for the synthesis of tpy end-modified polystyrenes (PSs), which is evident from the kinetic study as well as ^1^H NMR and gel permeation chromatography (GPC) analyses. Subsequently, the well-defined tpy-functionalized PSs obtained after decomplexation are successfully applied to the construction of metallo-supramolecular diblock copolymers (P3HT-Zn-PS), where the complexation and self-assembly characteristics are investigated by diffusion ordered spectroscopy (DOSY) NMR experiments.

## 2. Materials and Methods

### 2.1. Materials

Styrene (Showa, 99%, Tokyo, Japan) and acetonitrile (Fischer, HPLC grade, Loughborough, UK) were distilled over CaH_2_ under reduced pressure. *N*,*N*,*N′*,*N″*,*N″*-Pentamethyldiethylenetriamine (PMDETA) (Acros, 98%, Morris Plains, NJ, USA) was distilled over KOH under reduced pressure, and then degassed by three freeze-pump-thaw cycles in a Schlenk tube before use. CuBr (Alfa Aesar, 99%, Ward Hill, MA, USA) was purified by stirring overnight in AcOH, filtered, washed with absolute EtOH and diethyl ether, and then dried under vacuum. Unless otherwise noted, reagents and solvents were used as received from Fisher Scientific (Loughborough, UK) and Sigma-Aldrich (St. Louis, MO, USA) without further purification. 4′-(4-Hydroxyphenyl)-2,2′:6′,2″-terpyridine (**1**) [43], 9-anthraceneboronic acid [44], tetrakis(triethylammonium) ethylenediaminetetraacetate (TEA-EDTA) [45], 2,5-dibromo-3-hexylthiophene [46], mono-brominated P3HT (**Br–P3HT**) [19], 6,6″-di(anthracen-9-yl)-4′-(4-methoxyphenyl)-2,2′:6′,2″-terpyridine (**L2**) [19], 4′-(4-methoxyphenyl)-2,2′:6′,2″-terpyridine (**L3**) [47], and (**L2**–Zn–**L3**) complex [48] were prepared according to the reported procedures.

### 2.2. Methods

^1^H, ^13^C, and DOSY NMR experiments were performed at 25 °C on a Varian Mercury NMR 400 spectrometer, where chemical shifts (*δ* in ppm) were determined with respect to the nondeuterated solvents as a reference. Gel permeation chromatography (GPC) was conducted on the instrument equipped with two columns (Shodex KF-803 and KF-804, Tokyo, Japan), a Waters 515 HPLC pump, and a differential refractive index detector (LabAlliance RI2000, New York, USA). Tetrahydrofuran (THF) mixed with tetrabutylammonium bromide (TBAB) (1 wt%) was utilized as an eluent at a flow rate of 1 mL min^−1^ at 40 °C [49]. The calibration curve was established by linear polystyrene standards. MALDI-TOF-MS measurements were conducted on a Bruker Autoflex Speed MALDI-TOF-MS (Bruker Daltonics, Billerica, MA, USA). The polystyrene samples for MALDI-TOF-MS measurements were prepared by mixing the polymer (5.0 mg mL^−1^), dithranol (DIT) (10 mg mL^−1^), and trifluoroacetic acid (TFA) (1%) in THF.

### 2.3. General Procedure for Complexation Reactions

To a CHCl_3_ solution (5 mL) of **PS_n_** (n = 19, 33, 85, 106, 161, and 235) and **P3HT_54_** in an equimolar ratio calculated from the corresponding number average molecular weights (*M*_n,NMR_), 1 Eq of Zn(OTf)_2_ in MeOH (5 mL) was added. After the reaction mixture was stirred at room temperature for 5 min, the solvent was evaporated under reduced pressure to give the corresponding diblock copolymers.

## 3. Results and Discussion

**Scheme 1:**
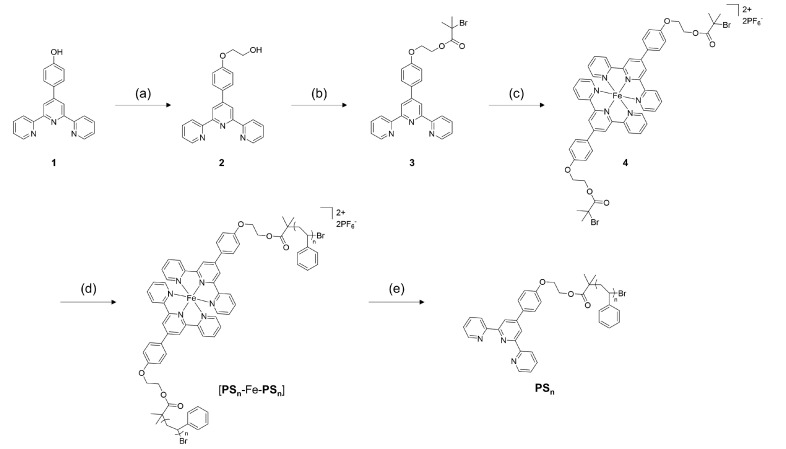
Synthesis of metallo-initiator **4** and **PS_n_**. *Reagents and conditions*: (**a**) K_2_CO_3_, dimethylformamide (DMF), 2-chloroethanol, 50 °C; (**b**) NEt_3_, *α*-bromoisobutyryl bromide, dichloromethane (DCM), 0 °C; (**c**) (1) FeCl_2_, MeOH/CHCl_3_, r.t., (2) NH_4_PF_6_; (**d**) styrene, CuBr, pentamethyldiethylenetriamine (PMDETA), MeCN, 110 °C; (**e**) tetrakis(triethylammonium) ethylenediaminetetraacetate (TEA-EDTA), DMF, r.t.

Inspired by the pioneering research of metallo-initiators for ATRP, we designed and synthesized an Fe(II) bis(tpy) complex bearing α-bromoester as a bifunctional metallo-initiator (**4**) (Scheme 1). The α-bromoester modified tpy-based initiator (**3**) was synthesized from the precursor 4’-(4-hydroxyphenyl)-tpy (**1**) in moderate yield. First, **1** was alkylated by 2-chloroethanol to give 4′-(4-(2-hydroxyethoxy)phenyl)-tpy (**2**). The following esterification was achieved by reaction of **2** with α-bromoisobutyryl bromide in the presence of triethylamine at room temperature to afford compound **3**. Subsequently, the complexation was conducted by adding a MeOH solution of FeCl_2_ (0.5 Eq) into a CHCl_3_ solution of ligand **3** (1 Eq) at room temperature, followed by counter-anion exchange with NH_4_PF_6_ (10 Eq), to yield **4** as a dark purple powder in quantitative yield. The suitable crystals for single-crystal X-ray crystallography were obtained by slow diffusion of diethyl ether into an MeCN solution of **4**. The structure of complex **4** was unequivocally established by its crystal structure (Figure S8), NMR spectroscopy (Figures S5 and S6) and ESI-MS (Figure S7).

Our attempts to conduct Cu(I)-mediated ATRP of styrene using the initiator **3** were unsuccessful possibly due to the strong chelating ability of tpy ligands acting as a catalyst poison. To investigate the controllability of ATRP of styrene initiated by the bifunctional metallo-initiator **4**, a kinetic study was performed via a typical ATRP protocol as follows. Initiator **4** (45.2 mg, 34.9 μmol) and CuBr (15.0 mg, 104.8 μmol) were added to a degassed Schlenk flask equipped with a stir bar. Subsequently, MeCN (1.3 mL) and styrene (3.6 mL, 31.4 mmol) were added into the flask, which was degassed by three freeze-pump-thaw cycles, followed by the addition of PMDETA (21.9 μL, 104.8 μmol), and then stirred at 110 °C. The polymerization solution was periodically sampled via a pre-degassed syringe to monitor the conversion of monomer by ^1^H NMR and calculate the theoretical molecular weight (*M*_n,theo_). The sampled solution was further treated with TEA-EDTA in dimethylformamide (DMF) for 1 day at room temperature to decomplex the <tpy–Fe(II)–tpy> junction to afford the tpy end-modified PSs. The corresponding *M*_n,GPC_ and PDI (*M*_w_/*M*_n_) were determined by GPC, and *M*_n,NMR_ was calculated from the ^1^H NMR peak integral ratios of polymerized styrene and terminal tpy.

**Figure 1:**
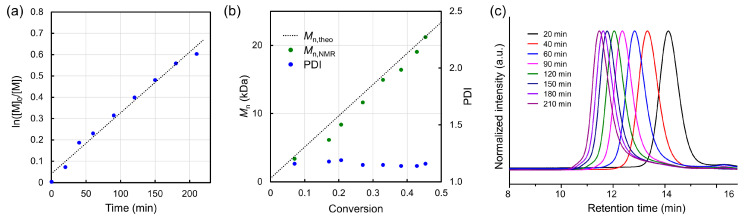
(**a**) Semilogarithmic kinetic plot, (**b**) *M*_n_ and *M*_w_/*M*_n_ versus conversion, and (**c**) gel permeation chromatography (GPC) traces for the atom transfer radical polymerization (ATRP) of styrene ([4]:[styrene]:[CuBr]:[PMDETA] = 1:900:3:3, styrene/MeCN = 3/1 (*v*/*v*), 110 °C).

The kinetic study on ATRP of styrene initiated by the metallo-initiator **4** was summarized in Figure 1. The semilogarithmic kinetic plot of ln([M]_0_/[M]) versus reaction time indicated the first-order radical polymerization process and the radical concentration was kept constant during the polymerization (Figure 1a). In addition, the experimental molecular weights (*M*_n,NMR_) were in good agreement with the theoretical ones (*M*_n,theo_) and linearly increased with respect to the monomer conversion (Figure 1b), implying the absence of significant chain transfer reactions. Moreover, the polydispersities were decreased with increasing conversion (Figure 1b), and a clear shift to higher molecular weights with a mono-distribution was evidenced by GPC traces (Figure 1c). These observations suggested that the well-controlled ATRP of styrene could be initiated by **4**.

**Figure 2:**
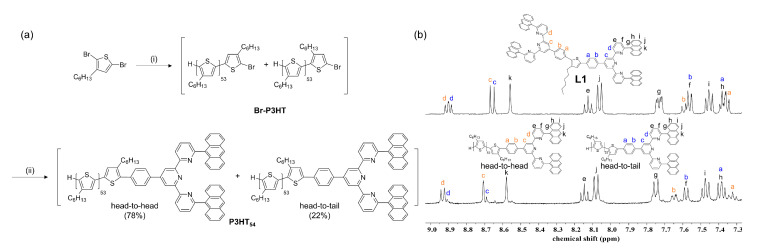
(**a**) Synthesis of **P3HT_54_**. *Reagents and conditions*: (i) (1) *t*-BuMgCl, LiCl, tetrahydrofuran (THF), (2) Ni(dppp)Cl_2_, THF, 0 °C; (ii) 6,6″-dianthracenyl-4′-(4-boronophenyl)tpy, Pd(PPh_3_)_4_, NaOH, THF/H_2_O (3/1, *v*/*v*), reflux. (**b**) ^1^H NMR spectra of ligand **L1** and **P3HT_54_**.

Based on the kinetic result of ATRP of styrene, a series of tpy-functionalized **PS_n_** (*n* = 19, 33, 85, 106, 161, and 235) with varying chain lengths was prepared via ATRP under optimized conditions (Table 1). The formation of well-defined **PS_n_** could be evident from the narrow molecular weight distributions and the consistency between *M*_n,NMR_ and *M*_n,GPC_. It is noteworthy that the terminal bromide at PS chain-ends susceptible to elimination easily led to formation of a double bond during MALDI-TOF-MS measurements [50]. Nevertheless, the high fidelity in the tpy chain-end functionality was verified by the corresponding MALDI-TOF-MS peaks (Figures S10, S12, S14, S16, and S18). On the other hand, the well-defined **P3HT_54_** (DP = 54, *M*_n,GPC_ = 8800 Da, *M*_w_/*M*_n_ = 1.23) end-functionalized with a 4-(4′-(6,6″-dianthracenylterpyridyl))phenyl group was obtained through the Suzuki–Miyaura coupling reaction of mono-brominated P3HT (**Br–P3HT**) with 6,6″-dianthracenyl-4′-(4-boronophenyl)tpy (Figure 2a) [19]. Notably, **Br–P3HT** prepared by Grignard metathesis (GRIM) polymerization method possessed two isomeric chain-end structures, i.e., head-to-head and head-to-tail orientations, which could not be differentiated by the ^1^H NMR spectrum of **Br–P3HT** but clearly seen in that of **P3HT_54_** (Figure 2b) [51,52]. Therefore, the 3-hexylthiophene coupled with two 6,6″-dianthracenyl-substituted tpys (**L1**) was synthesized as a model compound to ensure the proper ^1^H NMR assignments. The two sets of tpy signals of **L1** corresponded to two types of chain-end connections, and the chain-end head-to-tail content of **P3HT_54_** was estimated to be 22% (Figure S26). The single molecular weight distribution in the MALDI-TOF-MS spectra (Figure S27) strongly supported the high chain-end functionality for **P3HT_54_**.

**Scheme 2:**
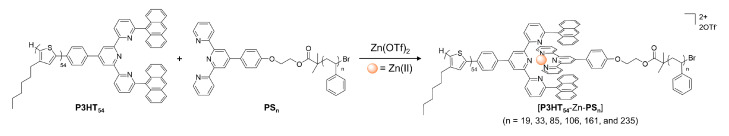
Construction of metallo-supramolecular diblock copolymers [**P3HT_54_**–Zn–**PS_n_**].

**Figure 3:**
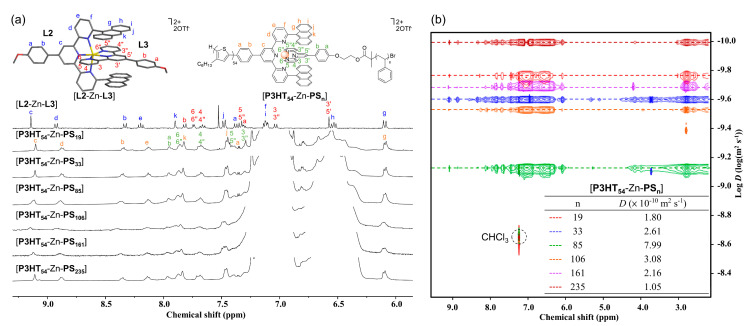
(**a**) ^1^H NMR spectra of [**L2**–Zn–**L3**] and [**P3HT_54_**–Zn–**PS_n_**] (n = 19, 33, 85, 106, 161, and 235). The upper left figure shows the X-ray crystal structure of [**L2**–Zn–**L3**] (gray, carbon; blue, nitrogen; red, oxygen; yellow, zinc). Hydrogen atoms and OTf^−^ ions are omitted for clarity. (**b**) Stacked diffusion ordered spectroscopy (DOSY) spectra of [**P3HT_54_**–Zn–**PS_n_**]. The inset table shows the corresponding diffusion coefficients determined with respect to the *D* of CHCl_3_ (2.33 × 10^−9^ m^2^ s^−1^) at 298 K [56].

We have demonstrated that the complementary complexation between 6,6″-substituted and unsubstituted tpy ligands with Zn(II) under ambient conditions could be applied to construction of the metallo-supramolecular diblock copolymers from two distinct tpy-modified homopolymers [19,48]. In the ligand design, the bulky 9-anthracenyl substituents effectively decelerated the formation rate of homoleptic complexes. Moreover, the X-ray single-crystal structure of [**L2**–Zn–**L3**] (Figure 3a) exhibited the π–π interactions between unsubstituted **L3** and two anthracenyl substituents to facilitate the formation of heteroleptic complexes. Consequently, a series of metallo-supramolecular diblock copolymers of [**P3HT_54_**–Zn–**PS_n_**] (*n* = 19, 33, 85, 106, 161, and 235) could be readily constructed from homopolymers **PS_n_** and **P3HT_54_** in the presence of Zn(II) ions (Scheme 2). Due to the labile coordination bonds, the intact copolymers could not be detected by MALDI-TOF-MS [53]. Hence, the resultant diblock copolymers were characterized by ^1^H NMR experiments (Figure 3a). The ^1^H NMR spectra of [**P3HT_54_**–Zn–**PS_n_**] strongly supported the formation of the desired heteroleptic junctions between **P3HT_54_** and **PS_n_** as compared with that of the model complex [**L2**–Zn–**L3**]. In addition, the diffusion ordered spectroscopy (DOSY) of [**P3HT_54_**–Zn–**PS_n_**] (5 mg mL^−1^ in CDCl_3_) revealed all the relevant ^1^H NMR resonances have the identical diffusion coefficients (*D*) for each copolymer (Figure 3b), implying the single distribution of hydrodynamic radii in solution [54]. Accordingly, as the chain length of the PS segment was decreased, the diffusion coefficient was increased for [**P3HT_54_**–Zn–**PS_235_**] (*D* = 1.05 ×10^−10^ m^2^ s^−1^), [**P3HT_54_**–Zn–**PS_161_**] (*D* = 2.16 × 10^−10^ m^2^ s^−1^), [**P3HT_54_**–Zn–**PS_106_**] (*D* = 3.08 × 10^−10^ m^2^ s^−1^), and [**P3HT_54_**–Zn–**PS_85_**] (*D* = 7.99 × 10^−10^ m^2^ s^−1^), due to the shrinkage in molecular size. However, [**P3HT_54_**–Zn–**PS_19_**] (*D* = 1.80 × 10^−10^ m^2^ s^−1^) and [**P3HT_54_**–Zn–**PS_33_**] (*D* = 2.61 × 10^−10^ m^2^ s^−1^) showed much smaller diffusion coefficients than expected, presumably because of the severe intermolecular aggregation, where the shorter PS length attenuated the interference in the assembly of P3HT segments [55].

## 4. Conclusions

A series of tpy end-modified polystyrenes with controllable molecular weights and narrow polydispersities was successfully prepared using a bifunctional tpy-based metallo-initiator via ATRP and subsequent decomplexation. Based on the pre-designed complementary ligand pairing, metallo-supramolecular diblock copolymers (P3HT-*b*-PS) were readily constructed from the well-defined P3HT and PS homopolymers end-functionalized with 6,6″-dianthracenyl-substituted tpy and unsubstituted tpy, respectively, upon the addition of Zn(II) ions. The DOSY NMR analysis not only supported the formation of the expected copolymers [P3HT–Zn–PS], but also revealed that the PS chain length would influence the assembly of P3HT segments in solution. We anticipate that the ATRP protocol using tpy-based metallo-initiators along with the complementary ligand pair will provide facile access to construction of various copolymers with enhanced topological diversity and complexity.

## Figures and Tables

**Table 1:** Results and optimized conditions for ATRP of styrene using **4**.

Entry	[4]:[styrene]:[CuBr]:[PMDETA] ^a^	Conversion (%)	*M*_n,NMR_^b^	DP ^b^	*M*_n,GPC_^c^	*M*_w_/*M*_n_^c^
**PS_19_**	1:100:3:3	31	2700	19	1900	1.14
**PS_33_**	1:200:3:3	48	4000	33	3600	1.13
**PS_85_**	1:200:3:3	55	9000	85	7900	1.21
**PS_106_**	1:400:3:3	46	11,100	106	11,900	1.14
**PS_161_**	1:800:3:3	33	16,700	161	16,200	1.17
**PS_235_**	1:800:3:3	64	24,500	235	22,000	1.26

^a^ Styrene/MeCN = 3/1 (*v*/*v*) for **PS_19_**, **PS_33_**, **PS_85_**, **PS_106_**, and **PS_161_**. **PS_235_** was obtained from the bulk polymerization. ^b^ *M*_n,NMR_ and degree of polymerization (DP) were calculated by ^1^H NMR. ^c^ *M*_n,GPC_ and *M*_w_/*M*_n_ were determined by GPC.

## Supplementary Materials

The following are available online at https://www.mdpi.com/2073-4360/12/12/2842/s1. Scheme S1. Synthesis of metallo-initiator **4**; Figures S1–S6. NMR spectra of compounds **2**-**4**; Figure S7. ESI-MS spectrum of **4**; Figure S8. X-ray crystal structure of **4**; Scheme S2. Synthesis of tpy-functionalized polystyrene **PS_n_** (n = 19, 33, 85, 106, 161, and 235) from the metallo-initiator **4**; Figures S9–S20. NMR spectra and MALDI-TOF-MS spectra of **PS_n_**; Figure S21. GPC traces of **PS_n_**; Scheme S3. Synthesis of **P3HT_54_**; Figures S22–S25. NMR spectra of compounds **6** and **7**; Figures S26–28. NMR, MALDI-TOF-MS, and GPC trace of **P3HT_54_**; Scheme S4. Synthesis of **L1**; Figures S29–S31. NMR spectra of **L1**; Table S1 and S2. Crystal data and experimental details for **4** and [**L2**–Zn–**L3**].
