# Supplementary material for: Synthesis of Terpyridine End-Modified Polystyrenes through ATRP for Facile Construction of Metallo-Supramolecular P3HT-b-PS Diblock Copolymers
Source: Polymers (Basel). 2020 Nov 29;12(12):2842. doi: 10.3390/polym12122842 (PMC7760035; doi:10.3390/polym12122842)
Supplement: Supplementary file 1 [file polymers-12-02842-s001.zip › MDPI_SI_R1.pdf]

Supporting Information

Synthesis of Terpyridine End-modified Polystyrenes through ATRP for Facile Construction of Metallo-supramolecular P3HT-*b*-PS Diblock Copolymers

Tsung-Han Tu and Yi-Tsu Chan*

Department of Chemistry, National Taiwan University, Taipei 10617, Taiwan; d02223101@ntu.edu.tw (T.-H.T.)
* Correspondence: ytchan@ntu.edu.tw (Y.-T.C.)

Table of Contents

1. Synthesis and Characterization of Metallo-initiator for ATRP....................... S2
2. Synthesis and Characterization of TPY-Functionalized PS S6
3. Synthesis and Characterization of **P3HT$_{54}$** S12
4. Crystal Data S18
References S20

1. Synthesis and Characterization of Metallo-initiator for ATRP

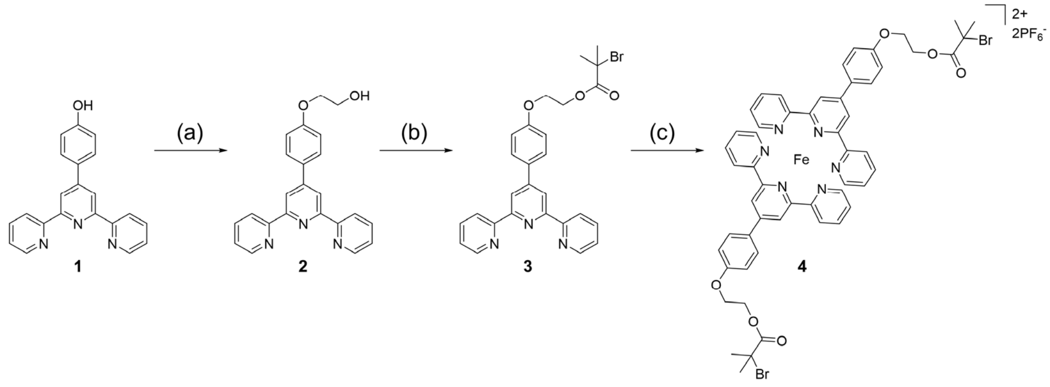

Scheme S1. Synthesis of metallo-initiator **4**. *Reagents and conditions*: (a) K_2CO_3, DMF, 2-chloroethanol, 50 °C; (b) NEt_3, α-bromoisobutyryl bromide, DCM, 0 °C; (c) 1) $FeCl_2$, MeOH/$CHCl_3$, r.t., 2) NH_4PF_6.

Compound 2. To a DMF (45 mL) solution of **1** (1.9 g, 5.8 mmol) and K_2CO_3 (4.8 g, 34.8 mmol), 2-chloroethanol (778.4 µL, 11.6 mmol) was added at room temperature. After being stirred at 50 °C for 18 h, the reaction mixture was extracted with ethyl acetate, and the combined organic extract was washed with H_2O, dried over anhydrous $MgSO_4$, and then evaporated to dryness under reduced pressure. The crude product was recrystallized from MeOH to give **2** as a white solid (1.1 g, 3.0 mmol) in 51% yield. ^{1}H NMR (400 MHz, $CDCl_3$): δ (ppm) 8.71 (d, *J* = 4.7 Hz, 2H), 8.69 (s, 2H), 8.65 (d, *J* = 8.0, 2H), 7.82–7.90 (m, 4H), 7.34 (dd, *J* = 7.7, 4.6 Hz, 2H), 7.03 (d, *J* = 8.2 Hz, 2H), 4.15 (t, *J* = 4.4 Hz, 2H), and 4.00 (td, *J* = 4.7, 4.6 Hz, 2H). ^{13}C NMR (100 MHz, $CDCl_3$): δ (ppm) 159.53, 156.34, 155.85, 149.66, 149.09, 136.86, 131.24, 128.61, 123.78, 121.37, 118.31, 114.91, 69.29, and 61.47. MALDI-TOF-MS: calcd for $C_{23}H_{20}N_3O_2$ $[M + H]^+$: *m*/*z* = 370.1556; found: 370.1678.

Compound 3. To a DCM (20 mL) solution of **2** (200.0 mg, 541.4 µmol) and NEt_3 (0.1 mL, 0.8 mmol), α-bromoisobutyryl bromide (86.8 µL, 0.7 mmol) was added at 0 °C. After being stirred at room temperature for 22 h, the reaction mixture was extracted with CH_2Cl_2 and H_2O. The combined organic extract was washed with H_2O, dried over anhydrous $MgSO_4$, and then evaporated to dryness under reduced pressure. The crude product was recrystallized from MeOH to give **3** as a white solid (227.3 mg, 438.5 µmol) in 81% yield. ^{1}H NMR (400 MHz, $CDCl_3$): δ (ppm) 8.73 (d, *J* = 4.6, 2H), 8.70 (s, 2H), 8.67 (d, *J* = 8.0, 2H), 7.86 (m, 4H), 7.35 (dd, *J* = 7.6, 5.4 Hz, 2H), 7.03 (d, *J* = 8.8 Hz, 2H), 4.57 (t, *J* = 4.9, 2H), 4.31 (t, *J* = 4.8, 2H), and 1.94 (s, 6H). ^{13}C NMR (100 MHz, $CDCl_3$): δ (ppm) 171.69, 159.38, 156.31, 155.84, 149.61, 149.08, 136.87, 131.38, 128.60, 123.78, 121.37, 118.31, 115.08, 65.73, 64.17, 55.48, and 30.73. MALDI-TOF-MS: calcd for $C_{27}H_{25}BrN_3O_3$ $[M + H]^+$: *m*/*z* = 518.1079; found: 518.1349.

Compound 4. To a $CHCl_3$ (5.0 mL) solution of **3** (50.0 mg, 96.4 µmol), $FeCl_2$ (6.1 mg, 48.2 µmol) in MeOH (5.0 mL) was added. After being stirred at room temperature for 30 min, an excess amount of NH_4PF_6 (157.1 mg, 964.0 µmol) was added to precipitate the counterion-exchanged complex, and then the mixture was stirred for an additional 30 min. The precipitate was filtered, washed with H_2O, and dried *in vacuo* to give **4** as a dark purple powder (66.6 mg, 48.1 µmol) in 99% yield. ^{1}H NMR (400 MHz, CD_3CN): δ (ppm) 9.08 (s, 4H), 8.54 (d, *J* = 8.0 Hz, 4H), 8.24 (d, *J* = 8.8 Hz, 4H), 7.84 (dd, *J* = 8.5, 7.0 Hz, 4H), 7.30 (d, *J* = 8.7 Hz, 4H), 7.13 (d, *J* = 5.5 Hz, 4H), 7.02 (dd, *J* = 7.3, 5.9 Hz, 4H), 4.57 (t, *J* = 4.5 Hz, 4H), 4.42 (t, *J* = 4.5 Hz, 4H), and 1.94 (s, 12H). ^{13}C NMR (100 MHz, CD_3CN): δ (ppm) 172.86, 162.39, 161.65, 159.61, 154.55, 151.37, 140.13,

130.91, 130.69, 128.69, 125.19, 122.40, 117.36, 67.79, 65.61, 57.90, and 31.51. ESI-MS: calcd for $C_{54}H_{48}Br_2FeN_6O_6$ [M − 2PF$_6$]$^{2+}$: m/z = 546.0699; found: 546.0760.

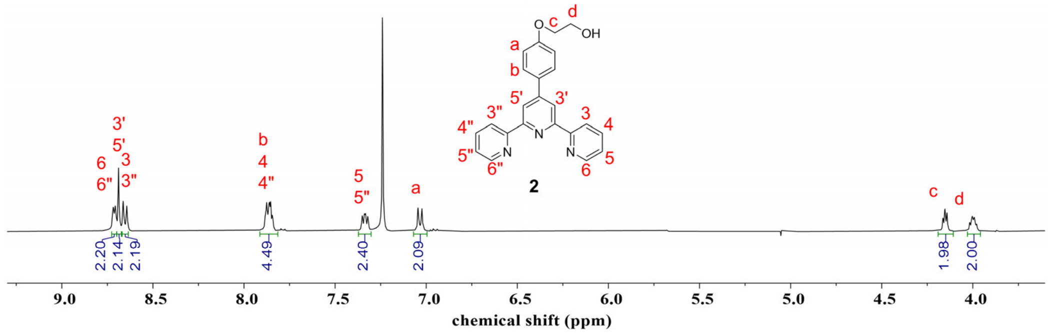

Figure S1. ^{1}H NMR spectrum of **2** in $CDCl_3$.

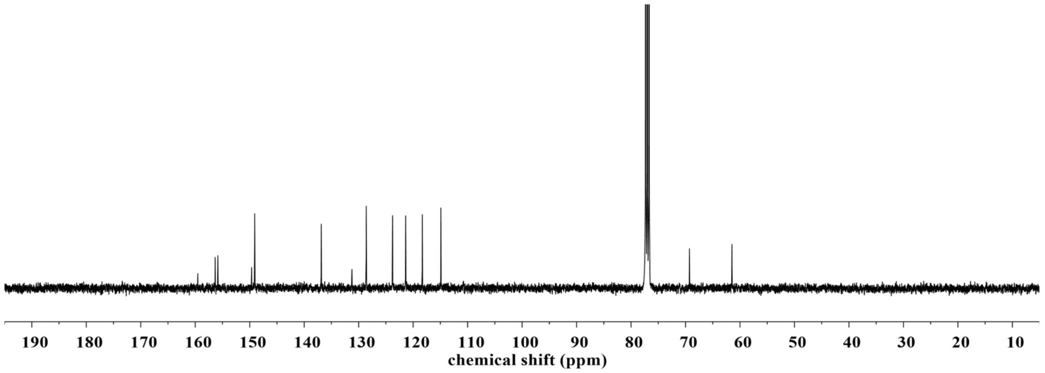

Figure S2. ^{13}C NMR spectrum of **2** in $CDCl_3$.

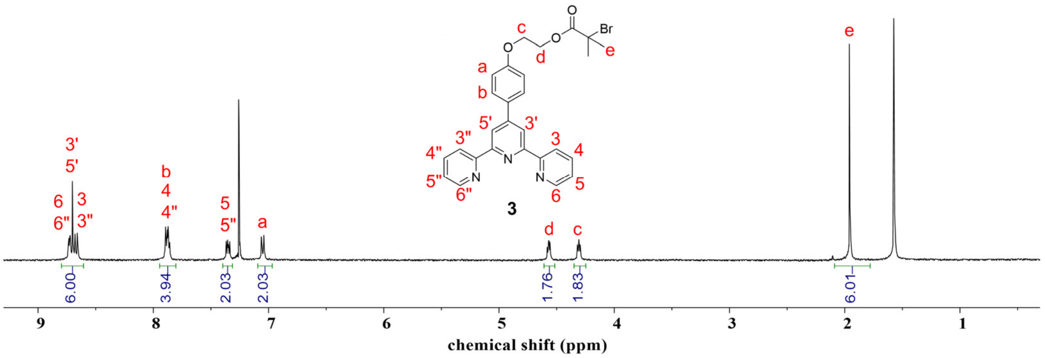

Figure S3. ^{1}H NMR spectrum of **3** in $CDCl_3$.

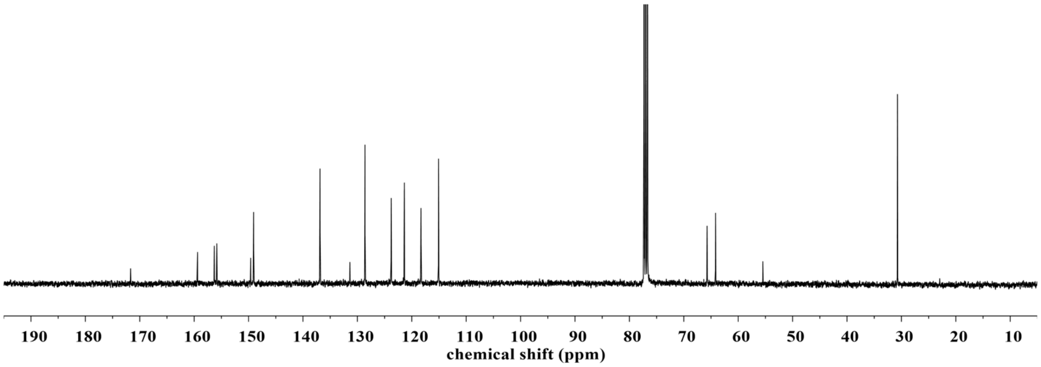

Figure S4. ^{13}C NMR spectrum of **3** in $CDCl_3$.

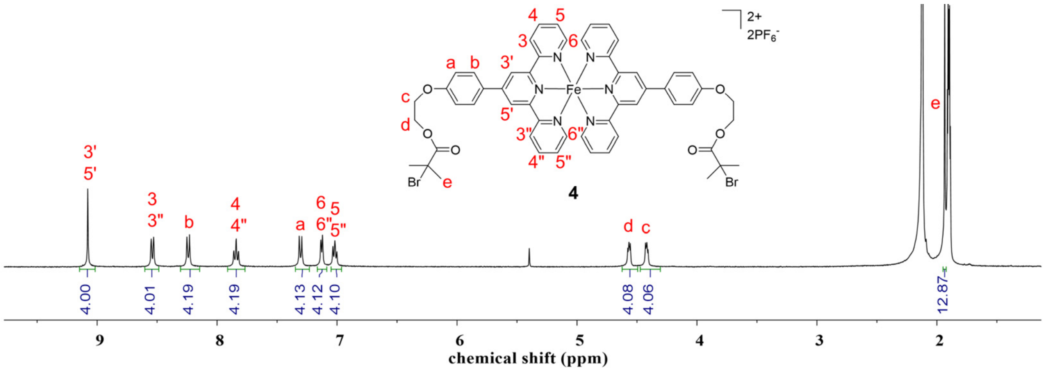

Figure S5. ^{1}H NMR spectrum of **4** in CD_3CN.

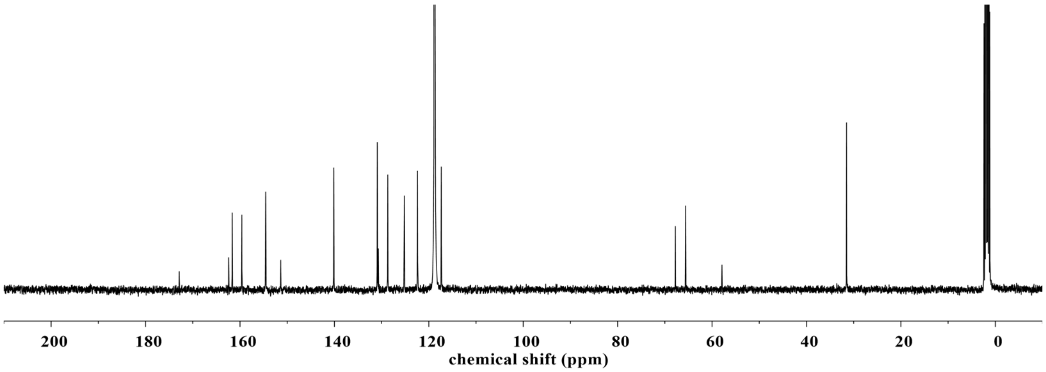

Figure S6. ^{13}C NMR spectrum of **4** in CD_3CN.

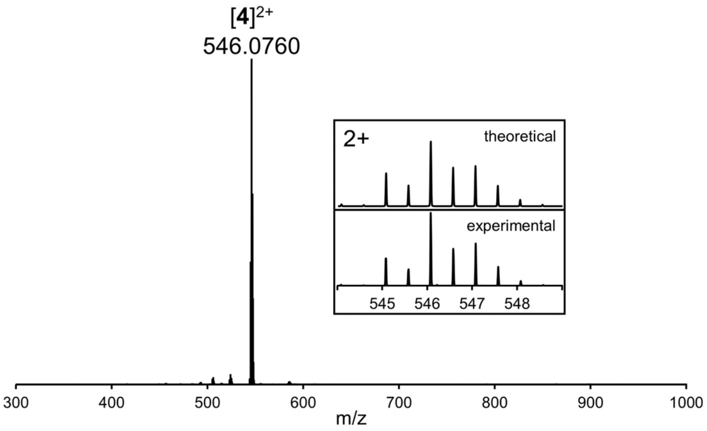

Figure S7. ESI-MS spectrum of **4**.

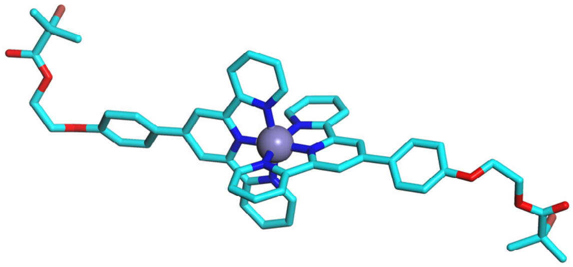

Figure S8. X-ray crystal structure of **4** (cyan, carbon; blue, nitrogen; red, oxygen; purple, iron; brown, bromine). Hydrogen atoms and PF_6^- ions are omitted for clarity.

2. Synthesis and Characterization of TPY-Functionalized PS

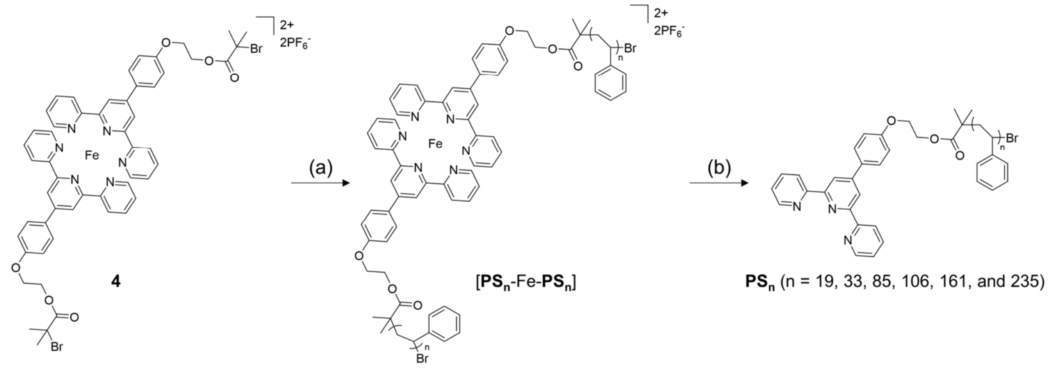

Scheme S2. Synthesis of tpy-functionalized polystyrene **PS_n** (n = 19, 33, 85, 106, 161, and 235) from the metallo-initiator **4**. *Reagents and conditions*: (a) styrene, CuBr, PMDETA, MeCN, 110 °C; (b) EDTA, DMF, 25 °C.

PS_{19}. To a degassed Schlenk flask containing CuBr (46.7 mg, 325.8 μmol) and **4** (150.1 mg, 108.6 μmol), the pre-degassed styrene (1.2 mL, 10.9 mmol) and MeCN (466.0 μL) were added. The flask was further degassed by three freeze-pump-thaw cycles, and then PMDETA (68.0 μL, 325.8 μmol) was added. After stirring at 110 °C for 60 min, the reaction mixture was cooled to room temperature and then exposed to air. An excess amount of TEA-EDTA in DMF was added to the mixture, which was subsequently stirred at 25 °C for 1 day. The reaction mixture was poured into MeOH and the precipitate was filtered and dried *in vacuo* to afford **PS_{19}** as a white powder (467.2 mg). ^{1}H NMR (400 MHz, $CDCl_3$): δ 8.75–8.61 (m, 6H), 7.92–7.79 (m, 4H), 7.36 (dd, J = 7.4, 5.1, 2H), 7.23–6.86 (br, 57H), 6.86–6.26 (br, 38H), 3.97–3.79 (br, 4H), and 2.28–1.14 (br, 57H). GPC: M_n = 1,900 Da, M_w/M_n = 1.14.

PS_{33}. By a similar procedure to that for **PS_{19}**, **PS_{33}** was obtained as a white powder (862.0 mg) from CuBr (40.3 mg, 280.8 μmol), **4** (129.4 mg, 93.6 μmol), styrene (2.1 mL, 18.7 mmol), MeCN (0.7 mL), and PMDETA (58.6 μL, 280.8 μmol). The polymerization was conducted at 110 °C for 2 h before quenched. ^{1}H NMR (400 MHz, $CDCl_3$): δ 8.75–8.63 (m, 6H), 7.90–7.80 (m, 4H), 7.33 (dd, J = 7.1, 4.9, 2H), 7.23–6.84 (br, 99H), 6.84–6.22 (br, 66H), 3.98–3.82 (br, 4H), and 2.10–1.17 (br, 99H). GPC: M_n = 3,600 Da, M_w/M_n = 1.13.

PS_{85}. By a similar procedure to that for **PS_{19}**, **PS_{85}** was obtained as a white powder (530.0 mg) from CuBr (20.3 mg, 141.3 μmol), **4** (65.1 mg, 47.1 μmol), styrene (1.1 mL, 9.4 mmol), MeCN (360 μL), and PMDETA (20.3 μL, 141.3 μmol). The polymerization was conducted at 110 °C for 8 h before quenched. ^{1}H NMR (400 MHz, $CDCl_3$): δ 8.86–8.66 (m, 6H), 8.02–7.86 (m, 4H), 7.49–7.37 (br, 2H), 7.23–6.85 (br, 255H), 6.85–6.25 (br, 170H), 3.98–3.81 (br, 4H), and 2.41–1.06 (br, 255H). GPC: M_n = 7,900 Da, M_w/M_n = 1.21.

PS_{106}. By a similar procedure to that for **PS_{19}**, **PS_{106}** was obtained as a white powder (463.1 mg) from CuBr (9.3 mg, 64.8 μmol), **4** (29.9 mg, 21.6 μmol), styrene (1.0 mL, 8.6 mmol), MeCN (498.6 μL), and PMDETA (13.5 μL, 64.8 μmol). The polymerization was conducted at 110 °C for 8 h before quenched. ^{1}H NMR (400 MHz, $CDCl_3$): δ 8.80–8.64 (m, 6H), 7.96–7.80 (m, 4H), 7.42–7.30 (br, 2H), 7.23–6.86 (br, 318H), 6.86–6.19 (br, 212H), 4.03–3.82 (br, 4H), and 2.33–1.12 (br, 318H). GPC: M_n = 11,900 Da, M_w/M_n = 1.14.

PS₁₆₁. By a similar procedure to that for **PS₁₉**, **PS₁₆₁** was obtained as a white powder (613.4 mg) from CuBr (9.3 mg, 64.8 μmol), **4** (29.8 mg, 21.6 μmol), styrene (2.0 mL, 17.3 mmol), MeCN (990.7 μL), and PMDETA (13.5 μL, 64.8 μmol). The polymerization was conducted at 110 °C for 16 h before quenched. ^{1}H NMR (400 MHz, $CDCl_3$): δ 8.79–8.66 (m, 6H), 7.94–7.84 (m, 4H), 7.40–7.30 (br, 2H), 7.30–6.88 (br, 483H), 6.88–6.24 (br, 322H), 3.95 (br, 4H), and 2.32–1.18 (br, 483H). GPC: M_n = 16,200 Da, M_w/M_n = 1.17.

PS₂₃₅. By a similar procedure to that for **PS₁₉**, **PS₂₃₅** was obtained as a white powder (1.0 g) from CuBr (9.3 mg, 64.8 μmol), **4** (29.8 mg, 21.6 μmol), styrene (2.0 mL, 17.3 mmol), and PMDETA (13.5 μL, 64.8 μmol). The reaction mixture was conducted at 110 °C for 3 h before quenched. ^{1}H NMR (400 MHz, $CDCl_3$): δ 8.82–8.65 (m, 6H), 7.97–7.83 (m, 4H), 7.40–7.33 (br, 2H), 7.23–6.86 (br, 705H), 6.86–6.25 (br, 470H), 3.99–3.85 (br, 4H), and 2.29–1.14 (br, 705H). GPC: M_n = 22,000 Da, M_w/M_n = 1.26.

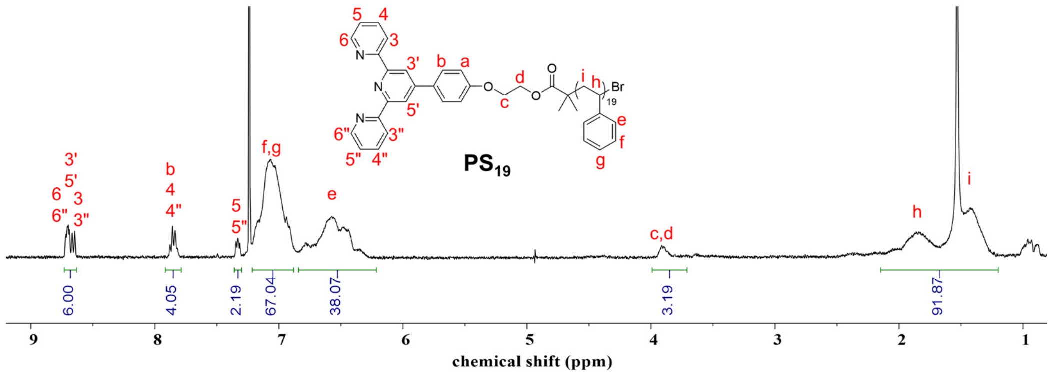

Figure S9. ^{1}H NMR spectrum of **PS₁₉** in $CDCl_3$.

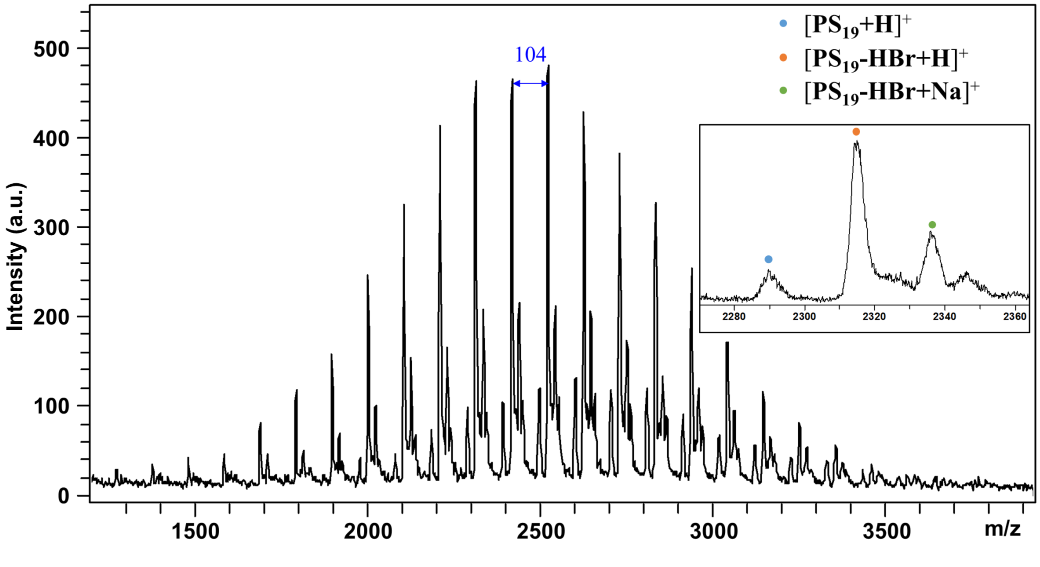

Figure S10. MALDI-TOF-MS spectrum of **PS₁₉**.

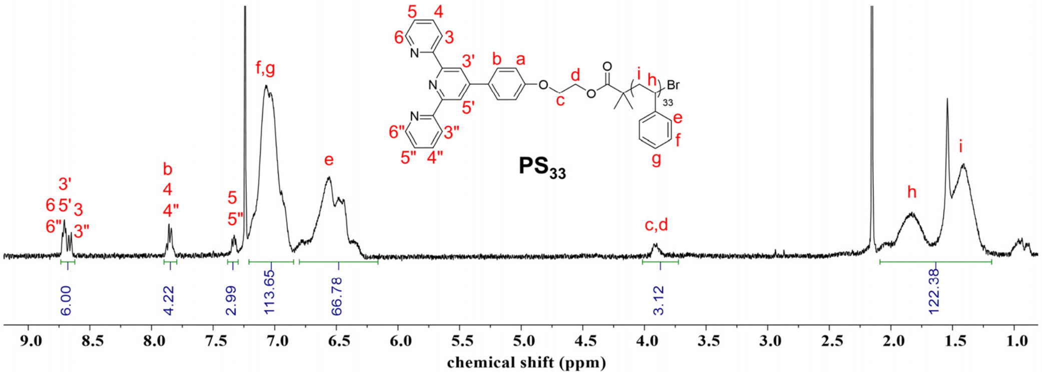

Figure S11. ¹H NMR spectrum of **PS**$_{33}$ in $CDCl_3$.

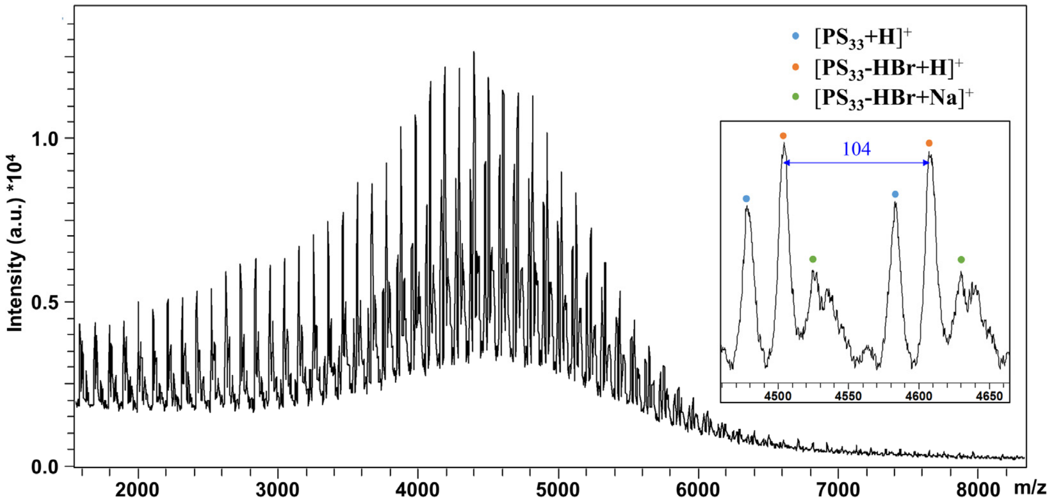

Figure S12. MALDI-TOF-MS spectrum of **PS**$_{33}$.

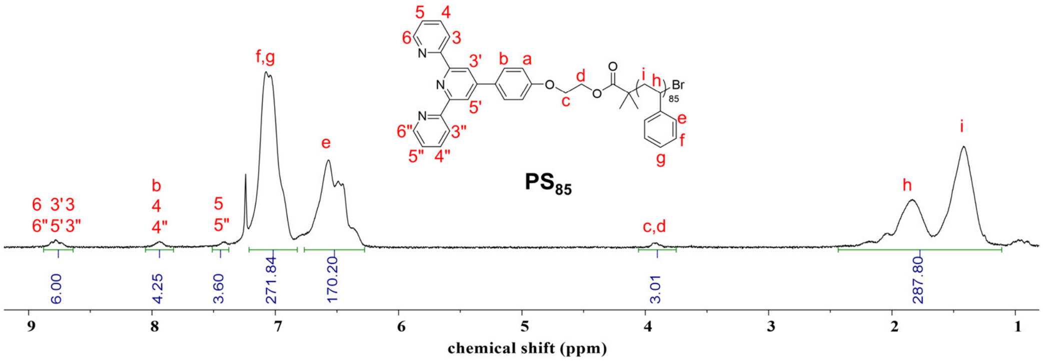

Figure S13. ¹H NMR spectrum of **PS**$_{85}$ in $CDCl_3$.

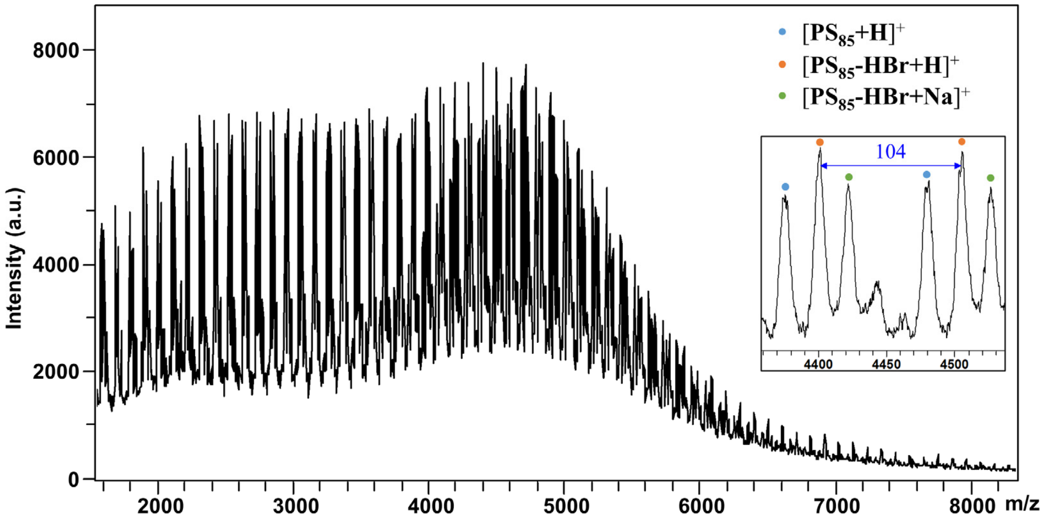

Figure S14. MALDI-TOF-MS spectrum of $\mathbf{PS_{85}}$.

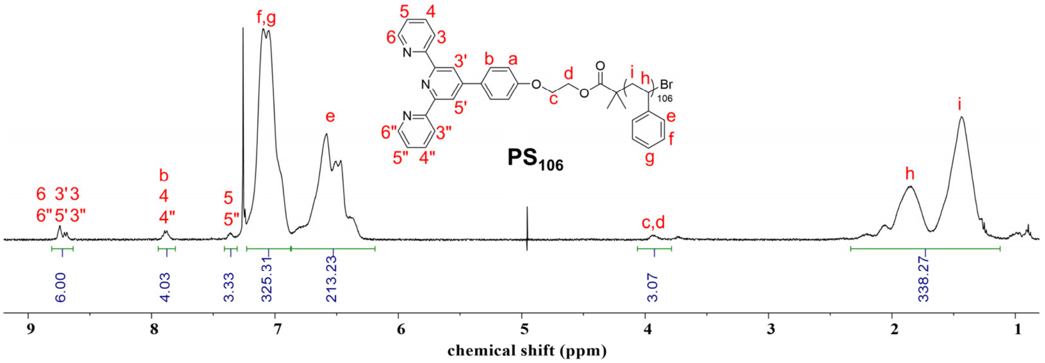

Figure S15. ^{1}H NMR spectrum of $\mathbf{PS_{106}}$ in $CDCl_3$.

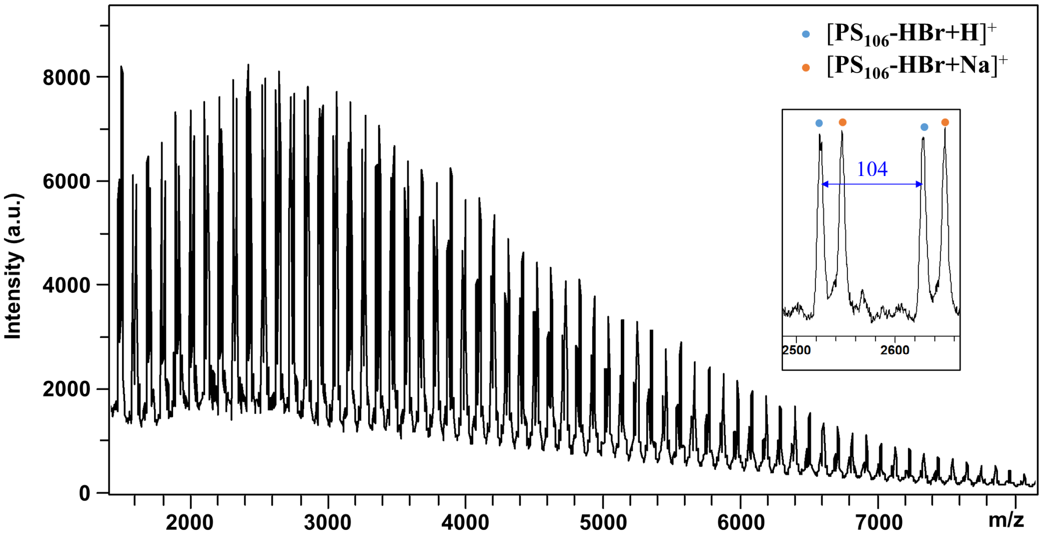

Figure S16. MALDI-TOF-MS spectrum of $\mathbf{PS_{106}}$.

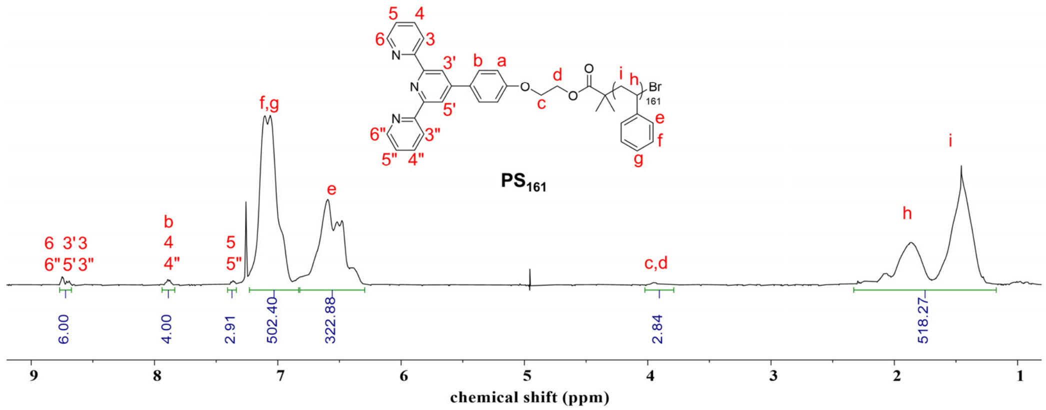

Figure S17. ^{1}H NMR spectrum of **PS**$_{161}$ in $CDCl_3$.

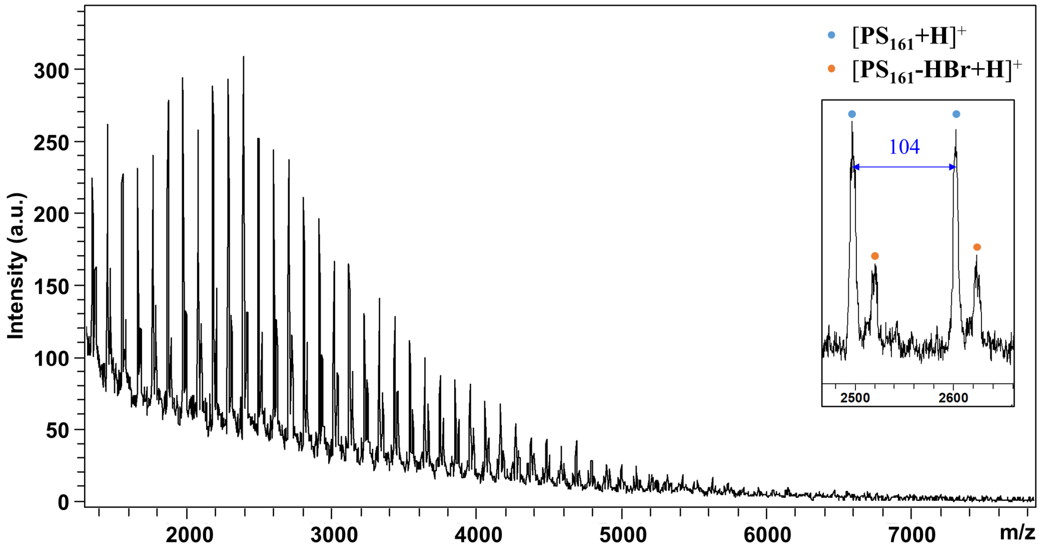

Figure S18. MALDI-TOF-MS spectrum of **PS**$_{161}$.

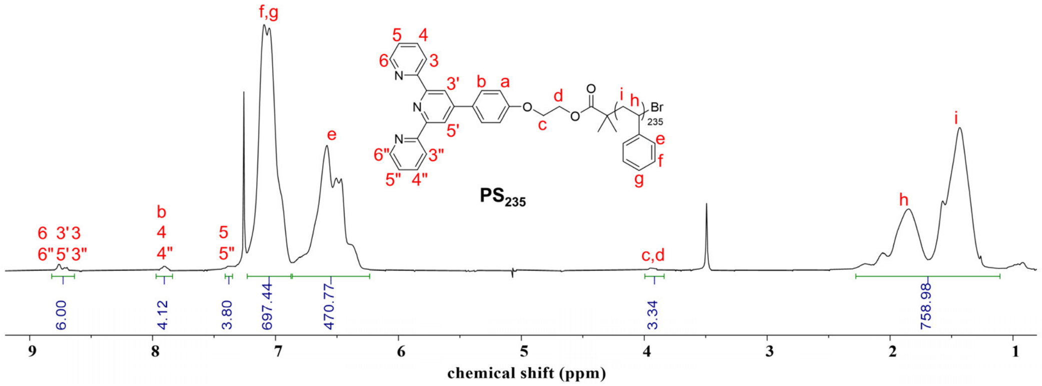

Figure S19. ^{1}H NMR spectrum of **PS**$_{235}$ in $CDCl_3$.

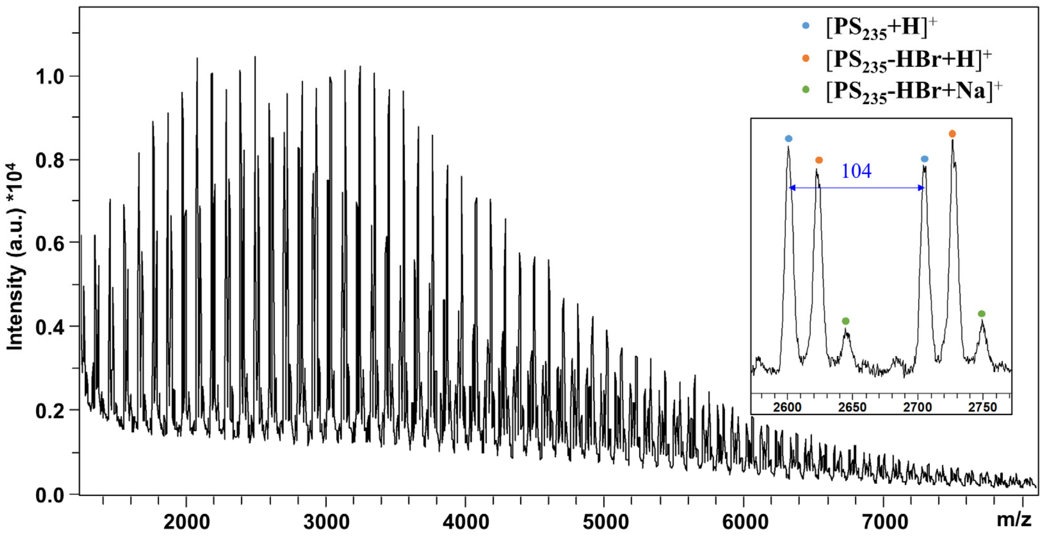

Figure S20. MALDI-TOF-MS spectrum of **PS**$_{235}$.

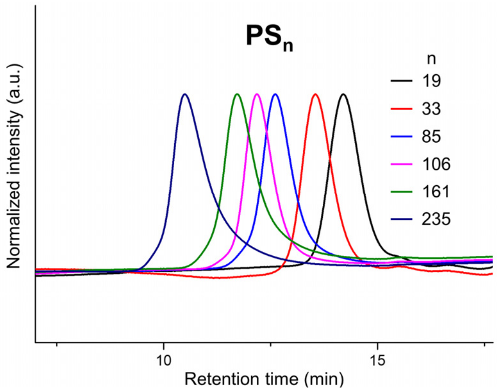

Figure S21. GPC traces of **PS**$_{19}$ (M_n = 1,900, M_w/M_n = 1.14), **PS**$_{33}$ (M_n = 3,600, M_w/M_n = 1.13), **PS**$_{85}$ (M_n = 7,900, M_w/M_n = 1.21), **PS**$_{106}$ (M_n = 11,900, M_w/M_n = 1.14), **PS**$_{161}$ (M_n = 16,200, M_w/M_n = 1.17), and **PS**$_{235}$ (M_n = 22,000, M_w/M_n = 1.26).

3. Synthesis and Characterization of P3HT$_{54}$

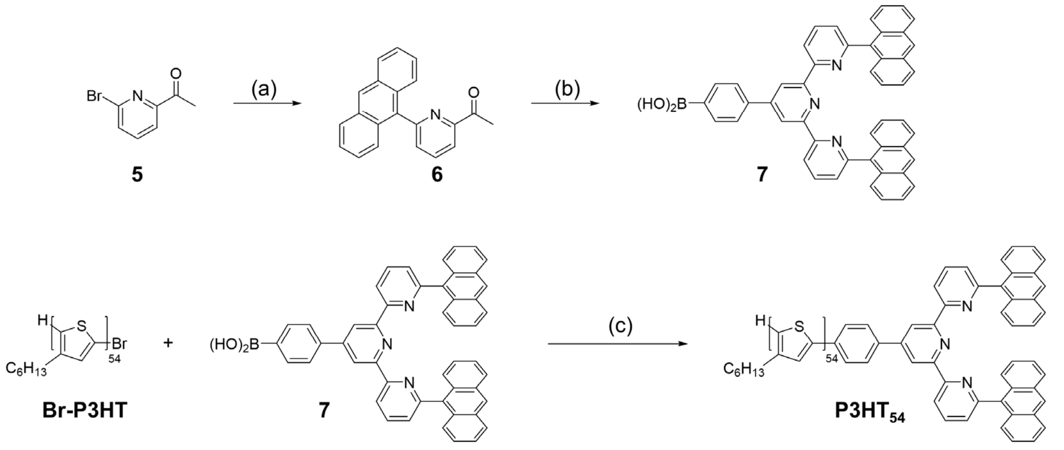

Scheme S3. Synthesis of **P3HT$_{54}$**. *Reagents and conditions*: (a) 9-anthraceneboronic acid, Na_2CO_3, $Pd(PPh_3)_4$, toluene/H_2O/*t*-BuOH (3/3/1, v/v/v), reflux; (b) 1) 4-formylphenylboronic acid, NaOH, EtOH, 25 °C, 2) NH_4OH, reflux; (c) $Pd(PPh_3)_4$, NaOH, THF/H_2O (3/1, v/v), reflux.

Compound 6. To a degassed two-neck flask containing **5** (15.0 g, 75.0 mmol), 9-anthraceneboronic acid (21.6 g, 97.5 mmol), and Na_2CO_3 (79.5 g, 750.0 mol), a mixed solvent (466.0 mL) of toluene/H_2O/*t*-BuOH (3/3/1, v/v/v) was added. After being purged with N_2 for 30 min, $Pd(PPh_3)_4$ (808.9 mg, 0.7 mmol) was added into the mixture, which was refluxed for 1 day under N_2. After cooling to 25 °C, the mixture was extracted with $CHCl_3$, and the combined organic extract was dried over anhydrous $MgSO_4$ and then evaporated to dryness under reduced pressure. The residue was recrystallized from MeOH to give **6** as a pale-yellow solid (20.0 g, 9.1 mmol) in 90% yield. ^{1}H NMR (400 MHz, $CDCl_3$): δ (ppm) 8.57 (s, 1H), 8.20 (d, J = 8.0 Hz, 1H), 8.10 – 8.00 (m, 3H), 7.65 (d, J = 8.1 Hz, 1H), 7.56 (d, J = 8.9 Hz, 2H), 7.47 (dd, J = 8.5, 7.5 Hz, 2H), 7.37 (dd, J = 8.7, 7.1 Hz, 2H), and 2.68 (s, 3H). ^{13}C NMR (100 MHz, $CDCl_3$): δ (ppm) 200.93, 157.90, 154.16, 137.31, 134.63, 131.62, 130.60, 130.24, 128.83, 128.17, 126.27, 126.01, 125.46, 120.44, and 26.25. MALDI-TOF-MS: calcd for $C_{21}H_{16}NO$ $[M + H]^+$: m/z = 298.1232; found: 298.1245.

Compound 7. To an EtOH (80 mL) solution of **6** (6.1 g, 20.5 mmol), NaOH (820.6 mg, 20.5 mmol) and 4-formylphenylboronic acid (1.4 g, 9.3 mmol) were successively added at 0 °C. After being stirred at 25 °C for 18 h, $NH_4OH_{(aq)}$ (28 wt%, 25 mL) was added into the mixture, which then was refluxed for 18 h. After cooling to 25 °C, the reaction mixture was filtered and washed with $CHCl_3$ to afford **7** as a yellow solid (4.0 g, 5.7 mmol) in 61% yield. ^{1}H NMR (400 MHz, CD_3OD/$CDCl_3$ = 4/1, v/v): δ (ppm) 8.90 (d, J = 7.8 Hz, 2H), 8.66 (s, 2H), 8.63 (s, 2H), 8.25 (dd, J = 7.8, 7.8 Hz, 2H), 8.13 (d, J = 8.4 Hz, 4H), 7.70 (d, J = 8.8 Hz, 4H), 7.64 (d, J = 7.2 Hz, 2H), and 7.54–7.38 (m, 10H). ^{13}C NMR (100 MHz, CD_3OD/$CDCl_3$ = 4/1, v/v): δ (ppm) 159.06, 158.58, 157.19, 153.17, 138.94, 138.85, 136.46, 136.39, 134.79, 132.90, 131.60, 129.83, 129.01, 128.41, 127.43, 127.29, 126.56, 121.99, 120.78, and 119.99. MALDI-TOF-MS: calcd for $C_{49}H_{33}BN_3O_2$ $[M + H]^+$: m/z = 706.2666; found: 706.2579.

P3HT$_{54}$. To a degassed two-neck flask containing **7** (302.1 mg, 428.5 μmol), mono-brominated **Br-P3HT** (2.0 g, 219.4 μmol, M_n = 9,100, M_w/M_n = 1.18), and NaOH (2.0 g, 50.0 mmol), a mixed solvent (170 mL) of THF/H_2O (3/1, v/v) was added. After being purged with N_2 for 30 min, $Pd(PPh_3)_4$ (30.5 mg, 26.4 μmol) was added into the mixture, which was then refluxed for 2 days under N_2. After cooling to room temperature, the mixture was extracted

with $CHCl_3$ and the combined organic extract was dried over $MgSO_4$ and then evaporated to dryness under reduced pressure. The residue was purified by precipitation from $CH_3OH/CHCl_3$ to give **P3HT$_{54}$** as a deep purple solid (2.0 g, 248.6 μmol, DP = 54) in 87% yield. ^1H NMR (400 MHz, $CDCl_3$) δ (ppm) 8.92 (m, 2H), 8.73 (s, 2H), 8.59 (s, 2H), 8.21 (m, 2H) 8.16 (m, 4H), 7.76 (m, 4H), 7.67 (m, 2H), 7.56 (s, 2H), 7.47 (s, 4H), 7.41 (s, 6H), 6.98 (s, 54H), 2.79 (s, 108H), 1.71 (s, 108H), 1.30 (d, 324H), and 0.92 (s, 108H). GPC: M_n = 8,800 Da, M_w/M_n = 1.23.

Isomeric structures of P3HT$_{54}$. The mono-brominated P3HT prepared by GRIM have two kinds of chain ends, namely head-to-head and head-to-tail orientations. The difference could be distinguished by the ^1H NMR spectra of **P3HT$_{54}$** [1]. The chain-end head-to-tail content is about 22% (Figure S26).

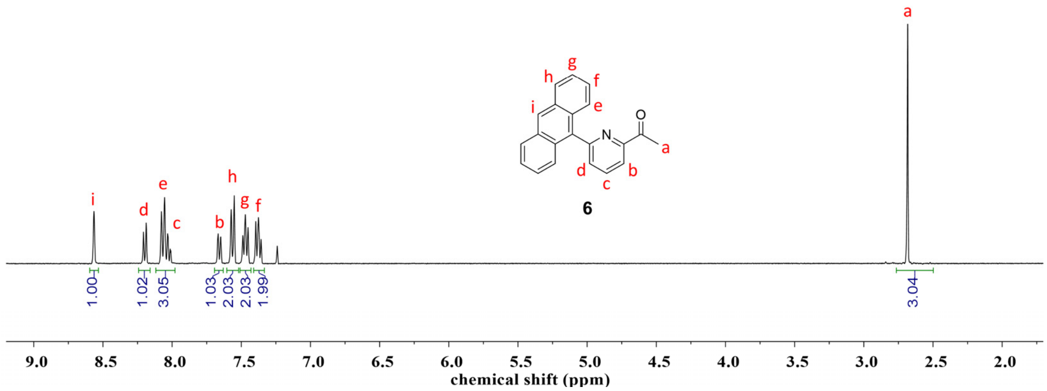

Figure S22. ^1H NMR spectrum of **6** in $CDCl_3$.

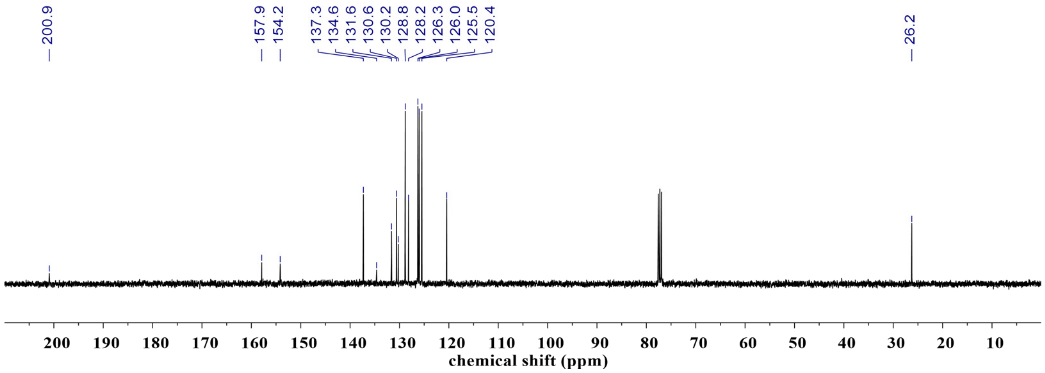

Figure S23. ^1H NMR spectrum of **6** in $CDCl_3$.

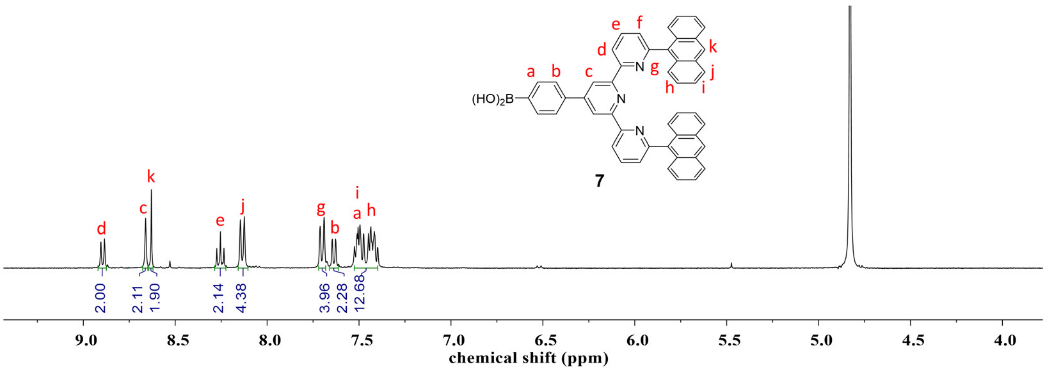

Figure S24. ^{1}H NMR spectrum of **7** in $CD_3OD/CDCl_3$ (4/1, v/v).

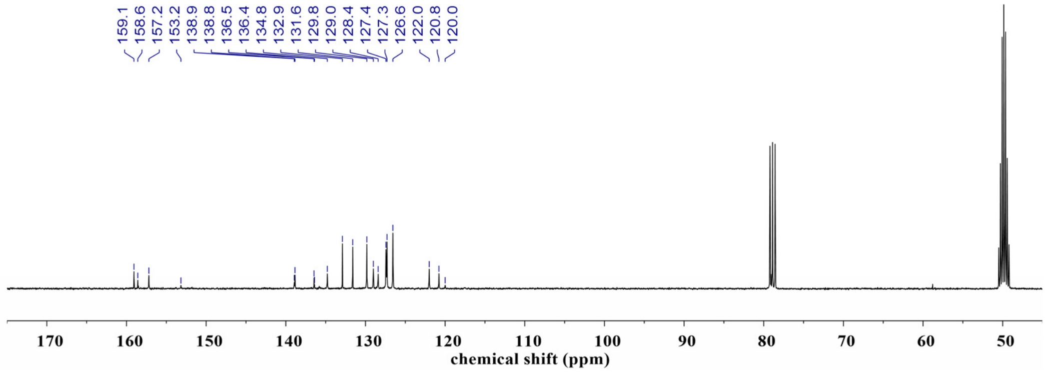

Figure S25. ^{1}H NMR spectrum of **7** in $CD_3OD/CDCl_3$ (4/1, v/v).

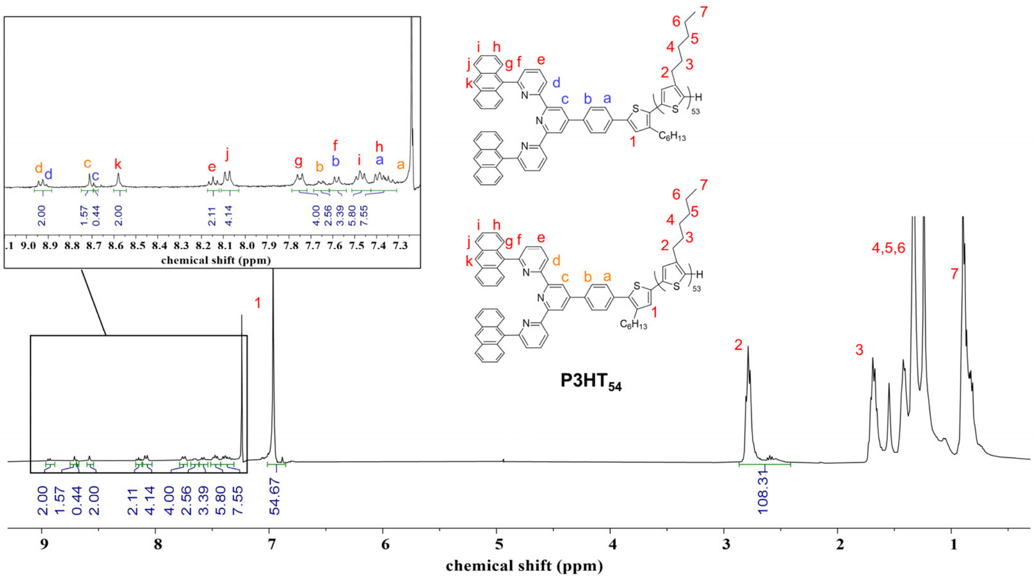

Figure S26. ^{1}H NMR spectrum of **P3HT$_{54}$**.

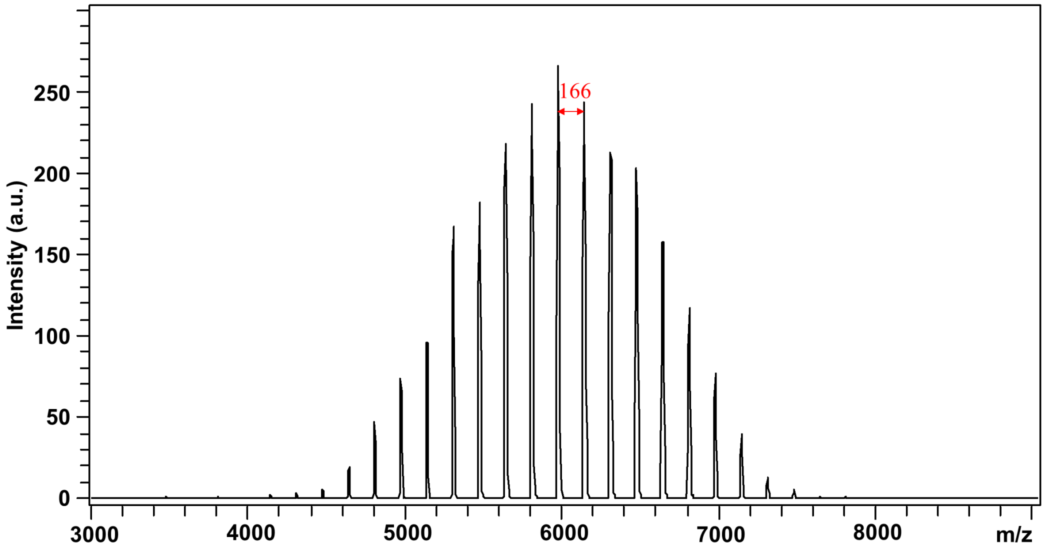

Figure S27. ^{1}H NMR spectrum of **P3HT**$_{54}$.

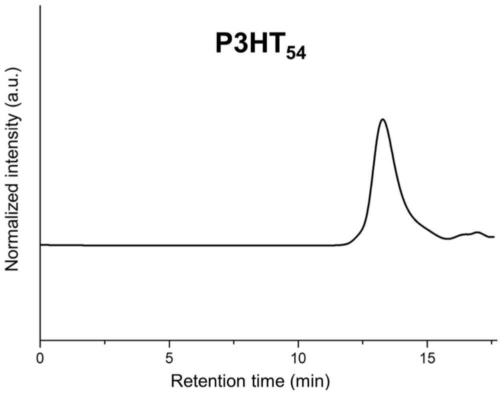

Figure S28. GPC trace of **P3HT**$_{54}$ (THF was used as eluent. M_n = 8,800 Da, M_w/M_n = 1.23).

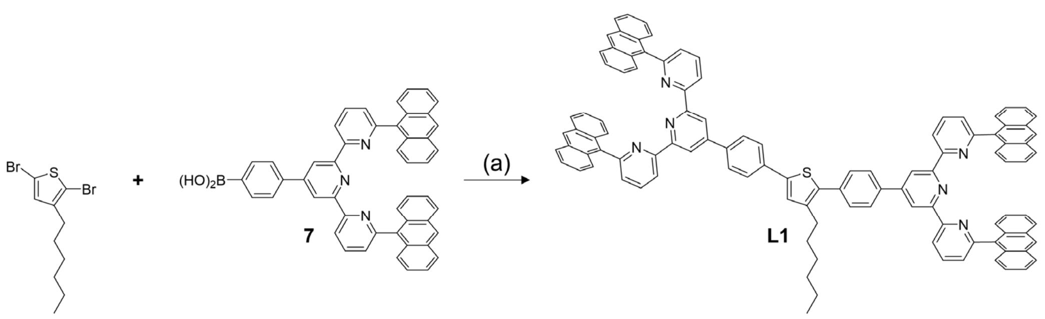

Scheme S4. Synthesis of **L1**. *Reagents and conditions*: (a) Na_2CO_3, $Pd(PPh_3)_4$, toluene/H_2O/*t*-BuOH (3/3/1, v/v/v), reflux.

Compound L1. To a degassed two-neck flask containing 2,5-dibromo-3-hexylthiophene (0.2 g, 0.6 mmol), **7** (1.3 g, 1.8 mmol), and Na_2CO_3 (2.2 g, 20.7 mmol), a mixed solvent (56 mL) of toluene/H_2O/*t*-BuOH (3/3/1, v/v/v) was added. After being purged with N_2 for 30 min, $Pd(PPh_3)_4$ (208.0 mg, 0.2 mmol) was added into the mixture, which was refluxed for 2 days under N_2. After cooling to 25 °C, the mixture was extracted with $CHCl_3$ and H_2O. The combined organic extract was dried over anhydrous $MgSO_4$ and then evaporated to dryness under reduced pressure. The residue was purified by column chromatography (Al_2O_3, CH_2Cl_2/hexane = 3/1) The crude was recrystallized from MeOH to give **L1** as a yellow solid (757.2 mg, 0.5 mmol) in 83% yield. ^1H NMR (400 MHz, $CDCl_3$): δ (ppm) 8.93–8.86 (m, 4H), 8.67 (s, 2H), 8.65 (s, 2H), 8.56 (s, 4H), 8.13 (dd, J = 8.5, 7.5 Hz, 4H), 8.06 (d, J = 8.1 Hz, 4H), 7.75–7.70 (m, 8H), 7.76–7.71 (m, 8H), 7.45 (dd, J = 8.6, 6.5 Hz, 8H), 7.41–7.32 (m, 10H), 7.25 (d, J = 8.6, 2H), 7.00 (s, 1H), 2.43 (t, J = 7.8 Hz, 2H), 1.46–1.37 (m, 2H), 1.31–1.22 (m, 6H), and 1.15–1.03 (m, 3H). ^{13}C NMR (100 MHz, $CDCl_3$): δ (ppm) 157.60, 157.58, 156.65, 156.61, 156.07, 149.56, 149.47, 141.43, 140.09, 137.03, 137.00, 136.88, 135.55, 135.49, 134.97, 134.61, 131.42, 130.14, 129.20, 128.43, 127.61, 127.53, 127.49, 127.45, 127.36, 127.33, 126.97, 126.32, 126.30, 125.98, 125.78, 125.76, 125.52, 125.15, 119.95, 119.92, 119.26, 119.10, 31.47, 30.72, 29.00, 28.79, 22.43, and 13.95. MALDI-TOF-MS: calcd for $C_{108}H_{75}N_6S$ $[M + H]^+$: m/z = 1487.5773; found: 1487.5695.

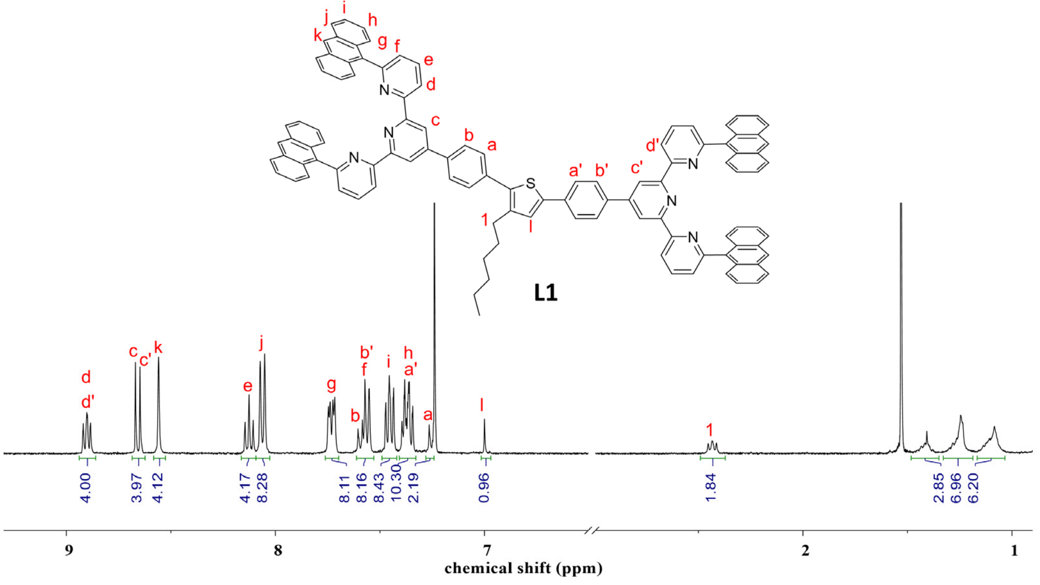

Figure S29. ^1H NMR spectrum of **L1** in $CDCl_3$.

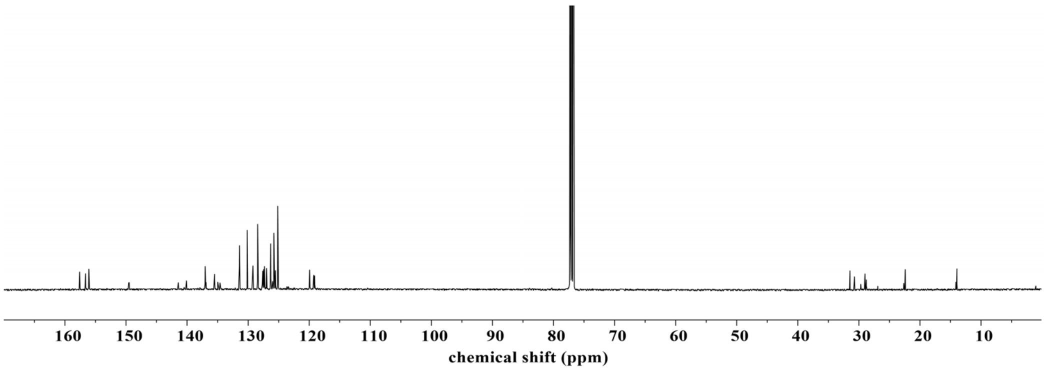

Figure S30. ^{13}C NMR spectrum of **L1** in $CDCl_3$.

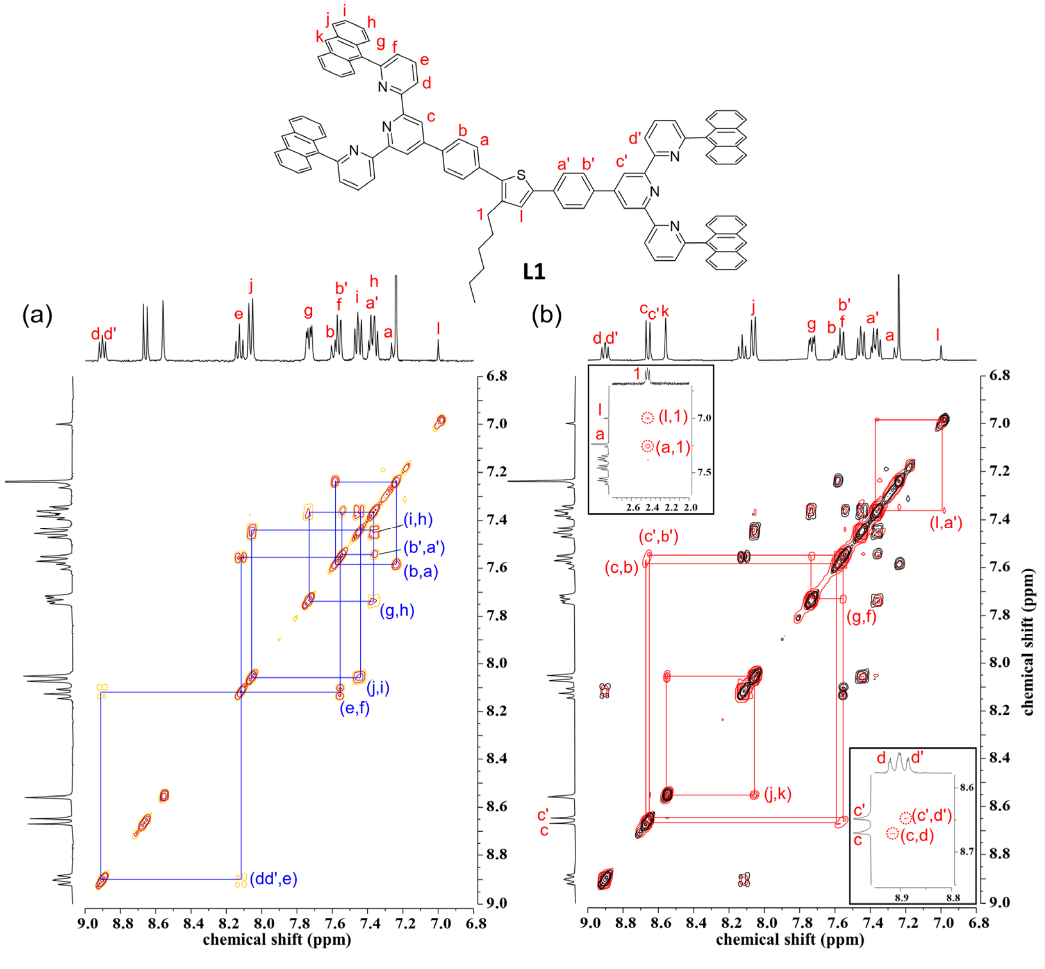

Figure S31. (a) COSY NMR spectrum of **L1** in $CDCl_3$. (b) Overlapped NOESY (red) and COSY (black) NMR spectra of **8** (insets: partial NOESY spectra).

4. **Crystal data**

Crystallographic data were acquired on an Oxford Diffraction Gemini A CCD diffractometer with graphite monochromated Mo-Kα radiation (λ = 0.71073 Å). The crystal structures of **4** and [**L2**-Zn-**L3**] were deposited at the Cambridge Crystallographic Data Center with the deposition numbers of CCDC 2043189 and 2043188, respectively.

Table S1. Crystal data and experimental details for metallo-initiator **4**.

Identification code	ic20571	
Empirical formula	$C_{54}H_{48}Br_2F_{12}FeN_6O_6P_2$	
Formula weight	1382.59	
Crystal system	Monoclinic	
Space group	Pn	
Unit cell dimensions	a = 16.473(2) Å	α = 90°.
	b = 9.0854(7) Å	β = 98.448(8)°.
	c = 37.973(3) Å	γ = 90°.
Volume	5621.5(9) Å^3	
Z	4	
F(000)	2784	
Density (calculated)	1.634 Mg m^{-3}	
Wavelength	0.71073 Å	
Cell parameters reflections used	4192	
Theta range for Cell parameters	3.1550 to 22.2550°.	
Absorption coefficient	1.842 mm^{-1}	
Temperature	100(2) K	
Crystal size	0.300 x 0.250 x 0.010 mm^3	
Data collection		
Diffractometer	Xcalibur, Atlas, Gemini	
Absorption correction	Semi-empirical from equivalents	
Max. and min. transmission	1.00000 and 0.68911	
No. of measured reflections	23625	
No. of independent reflections	14606 [R(int) = 0.0749]	
No. of observed [I>2_igma(I)]	7263	
Completeness to theta = 25.242°	99.7 %	
Theta range for data collection	3.136 to 27.499°.	
Refinement		
Final R indices [I>2sigma(I)]	R1 = 0.0914, wR2 = 0.2037	
R indices (all data)	R1 = 0.2073, wR2 = 0.2762	
Goodness-of-fit on F^2	1.277	
No. of reflections	14606	
No. of parameters	1538	

No. of restraints	519
Absolute structure parameter	0.19(2)
Largest diff. peak and hole	1.258 and -1.244 e.Å^{-3}

Table S2. Crystal data and experimental details for [**L2**-Zn-**L3**].

Identification code	ic20502	
Empirical formula	$C_{78}H_{60}F_6N_6O_9S_2Zn$	
Formula weight	1468.81	
Crystal system	Monoclinic	
Space group	Cc	
Unit cell dimensions	a = 24.443(4) Å	α = 90°.
	b = 17.210(3) Å	β = 124.58(3)°.
	c = 20.276(4) Å	γ = 90°.
Volume	7023(3) Å^3	
Z	4	
F(000)	3032	
Density (calculated)	1.389 Mg m^{-3}	
Wavelength	0.71073 Å	
Cell parameters reflections used	3806	
Theta range for Cell parameters	4.0900 to 26.5370°.	
Absorption coefficient	0.489 mm^{-1}	
Temperature	110(2) K	
Crystal size	0.20 x 0.15 x 0.10 mm^3	
Data collection		
Diffractometer	Xcalibur, Atlas, Gemini	
Absorption correction	Semi-empirical from equivalents	
Max. and min. transmission	1.00000 and 0.76530	
No. of measured reflections	16379	
No. of independent reflections	10909 [R(int) = 0.0415]	
No. of observed [I>2_igma(I)]	6767	
Completeness to theta = 25.242°	99.5 %	
Theta range for data collection	3.115 to 27.498°.	
Refinement		
Final R indices [I>2sigma(I)]	R1 = 0.0754, wR2 = 0.1860	
R indices (all data)	R1 = 0.1281, wR2 = 0.2309	
Goodness-of-fit on F^2	1.130	
No. of reflections	10909	
No. of parameters	919	

No. of restraints	50
Absolute structure parameter	0.015(13)
Largest diff. peak and hole	0.862 and -0.638 e.$Å^{-3}$

References

1. He, Y.-J.; Tu, T.-H.; Su, M.-K.; Yang, C.-W.; Kong, K.V.; Chan, Y.-T. Facile Construction of Metallo-Supramolecular Poly(3-hexylthiophene)-*block*-Poly(ethylene oxide) Diblock Copolymers via Complementary Coordination and Their Self-Assembled Nanostructures. *J. Am. Chem. Soc.* **2017**, *139*, 4218-4224, doi:10.1021/jacs.7b01010.
